# Annotated protein network analysis linking oral diseases

**DOI:** 10.6026/97320630018724

**Published:** 2022-08-31

**Authors:** Mukesh Kumar Sharma, Vivek Kumar Srivastav, Chetan Kumar Joshi, Mohan Kumar, Deepak Kumar Sharma

**Affiliations:** 1 Department of Biotechnology, Maharaj Vinayak Global University, Jaipur Rajasthan, India; 2 Department of Botany, Vishwa Bharti PG College, Sikar, Rajasthan India; 3 Department of Zoology, Govt. Science College, Sikar, Rajasthan, India; 4 Gyan Joyti College of Pharmacy and Nursing School, Hazaribagh, Jharkhand, India; 5 Department of Conservative and Endodontics, Jaipur Dental College, Jaipur, Rajasthan India

**Keywords:** Networking, STRING, oral cancer, bacteria, cystoscope

## Abstract

Oral cancer is becoming more common, and it threatens to be a serious worldwide medical issue. Hence, it is of interest to elucidate the networks between proteins and biologically active compounds, as well as their functional annotations, and cell signaling pathways. The online STRING software was used to create a molecular genetics interaction network named AZURIN on oral bacterial proteins. We also used the cystoscope software to identify 11 nodes and 16 edges with an average node order of 2.91. Thus, we document data on the interaction of protein networks with other proteins for identifying potential therapeutic drug candidates linked to oral disease.

## Background:

Head and neck tumors are one of the 10 most common types of cancer in the world. Oral squamous cell carcinoma (OSCC) in men is the most common cancer of all head and neck squamous cell carcinomas (HNSCC). It accounts for about 90% of oral malignancies and accounts for more than 300,000 newly diagnosed cases each year [1-3]. The molecular basis for aggressive OSCC growth and metastasis is still unknown. OSCCs often remain undetected at the high stages associated with high mortality and are therefore associated with high personal and social costs. The 5-year overall survival rate is estimated to be about 50% [3]. Therefore, reducing oral cancer mortality requires new targets for early detection and treatment of OSCC. In recent years, the identification of genes associated with complex diseases has become an important issue. Experimental approaches, such as B. genetic linkage association studies [4], expression profiling [5], and genome-wide association studies [6], are for genes at high relative risk for diseases such as cancer [7] and asthma [8]. It is successful in identifyingDiabetes [9] and hypertension [10]. However, disease heterogeneity, the complexity of finding genes at specific loci, and the associated costs have led to the development of various in-silico approaches to the identification of diseased genes. The correlation between a particular bacterium and various diseases, including cancer, is well known and well known. The role of bacteria in some types of cancer has been elucidated in more recent studies but remains unclear in many other studies [11]. Assessing the dynamics of a microbial population under a medical condition helps determine the mechanisms by which bacteria can be used to induce or develop cancer. Periodontal disease is caused by a bacterial infection of the periodontal tissue that causes gingival inflammation and periodontal disease. Periodontitis affects the gingival tissue but not the underlying tooth support structure. Periodontal disease, in contrast, it's an inflammatory disease that extends deep into the tissue and results in the loss of supporting connective tissue. Periodontitis can lead to loss of connective tissue and bone support and is the leading cause of tooth loss in adults [12]. Caries are the destruction of tooth structure by oral bacterial acids produced by microbial fermentation of leftover food [13]. Three characteristics of bacterial species are involved in biofilm formation, acid production, and caries development, including acid resistance [14]. Many acid-forming and acid-forming bacteria are involved in the carrier, including Streptococcus mutans and Streptococcus sorbinus (collectively known as mutans Streptococcus), as well as other acidic strains of lactobacillus and non-mutans streptococcus [15]. This study follows an observational study design aimed at screening for virulence factors in the oral microflora with these proteins ororal cancer and chewing tobacco complex pathogens. This can probably interact with certain proteins. The interaction of oral cancer protein cross-linking with oral mediator proteins and other proteins was analyzed using the STRING v.5 pipeline (Szklarcz) [16]. Therefore, it is of interest to document the annotated protein network analysis linking oral diseases.

## Methodology:

### Sources and selection of the targeting protein:

The source of the plant, the geographic location of the collection, the chemical structure, and the biological activity of the oral bacterial protein azurin and oral cancer cyclin B1 protein were obtained from bibliographic sources, The origin of the plant, the geographic location of the collection, the chemical structure and biological activity of the oral bacterial proteins azurin and oral cancer cyclin B1 proteins were obtained from bibliographic sources including major journals, masters and papers, chapters of natural product chemistry. The standards used are described by Kumar et al. [17] and Gupta et al. [18].

### Protein and compounds network interaction:

Extensive network analysis is used to identify functional connections between proteins and emphasize the biological importance of genes linked to enrichment pathways. The STRING and STITCH (version: 11.0) databases are global resources for predicting functional connections between proteins and cloud cluster networks and are used to study the interactions between proteins encoded by specific genes. Experiments to identify species using P proteins and chemicals as input gene sets are completed [19]. Study protein interactions (PPIs) between enriched genes and networks of interactions between them. A full score above 0.7 indicates a high level of confidence in the presence of significant interactions.

## Results and Discussion:

Protein-protein interactions are a central part of the cell network and are known to have many effects. Analyze the information flow network between all target proteins to determine the amount of information that flows between cytochrome proteins and other proteins. Use online STRING software to create a molecular genetic interaction network azulin on oral bacterial proteins, and use Cytoscape software to get 11 nodes, 16 edges, an average node order of 2.91, and visualization averages. I calculated. The local clustering factor is 0.876, the expected number of edges is 10, and the enrichment ppi has a p-value of 0.0553. Nodes, lines, and colors show the rationality of interactive networks (Figure 1 - see PDF) (Table 1 - see PDF).

For interleukin 8 in oral squamous cell carcinoma, a molecular genetic interaction network was obtained and the software Cytoscape was used to visualize the number of nodes 11, the number of edges 55, the average node order 10, and the values. It was made into. The local clustering factor is 1, the expected number of edges is 20, and the enrichment p-value is ppi 3.25e11 (Figure 2 - see PDF) (Table 3 - see PDF). Nodes, lines, and colors prove the rationality of interactive networks. Many experiments have shown that the gene is associated with protein expression. The results show that more residues than the cytochrome-protein cross-linking pathway are involved in protein signal intensity.

The result is represented by the color displayed in the forecast tree. A random machine learning approach [20] was used to build a reliable PPI network by combining co-movement scores obtained from metabolic partitions with detailed information suggesting physical links (Figure 2A - see PDF).This is because physically interacting proteins exert similar biological activity, co-express, and often have similar evolutionary conservation. Only protein pairs with solid biochemical evidence (at least 0.4 correlation rating) from the fractionated dataset were used, and other supporting features were implemented in this subset. Two additional measurements were recorded from biochemical fractional records that reflect reproducibility. Specifically, there are several fractionation experiments in which the co-migration assessment of the protein pair is at least 0.4, and several fractionation experiments with the largest peak in each migration profile. Duplicate protein pairs. Other evidence supporting functional associations is co-[deletion][21-23] interacting domains [24,25], co-evolution [26] and phenotypic records [26] (Table 3 - see PDF). Machine learning classifiers trained on a gold standard set containing only experimentally determined bacterialazulin proteins rubA2, rubA1, oprC, nirS, nirS, nirM, exaB, exaB, ccoQ2, ccoQ1, rubA1, azu, nirM, exaBand tested. (Table 2 - see PDF) is responsible for the annotated interactions in the STRING database [18]. Interactive network proteins such as CCL2, CCL3, CSF2, IL10, IL1A, IL1B, IL6, TNF and IL10 are shown among oral cancer proteins. Assessment of the relative contribution of each property to PPI prediction (measured by GiniScore) confirmed that the combined biochemical evidence had the greatest effect on classification (Table 4 - see PDF) and was associated with other functional associations. Compared to better co-mobility, it shows that it reflects information and is used to predict interactions.

## Conclusions:

We document preliminary data from a comprehensive analysis of the interaction of protein networks with other proteins that can be used as potential therapeutic drug candidates for the prevention of oral disease.
